# Development, testing, parameterisation, and calibration of a human PBK model for the plasticiser, di (2-ethylhexyl) adipate (DEHA) using *in silico*, *in vitro* and human biomonitoring data

**DOI:** 10.3389/fphar.2023.1165770

**Published:** 2023-03-23

**Authors:** Kevin McNally, Craig Sams, George Loizou

**Affiliations:** Health and Safety Executive, Harpur Hill, Buxton, United Kingdom

**Keywords:** plasticiser, DEHA, PBPK, *in silico*, reverse dosimetry

## Abstract

**Introduction:** A physiologically based biokinetic model for di (2-ethylhexyl) adipate (DEHA) based on a refined model for di-(2-propylheptyl) phthalate (DPHP) was developed to interpret the metabolism and biokinetics of DEHA following a single oral dosage of 50 mg to two male and two female volunteers.

**Methods:** The model was parameterized using *in vitro* and *in silico* methods such as, measured intrinsic hepatic clearance scaled from *in vitro* to *in vivo* and algorithmically predicted parameters such as plasma unbound fraction and tissue:blood partition coefficients (PCs). Calibration of the DEHA model was achieved using concentrations of specific downstream metabolites of DEHA excreted in urine. The total fractions of ingested DEHA eliminated as specific metabolites were estimated and were sufficient for interpreting the human biomonitoring data.

**Results:** The specific metabolites of DEHA, mono-2-ethyl-5-hydroxyhexyl adipate (5OH-MEHA), mono-2-ethyl-5-oxohexyl adipate (5oxo-MEHA), mono-5-carboxy-2-ethylpentyl adipate (5cx-MEPA) only accounted for ∼0.45% of the ingested DEHA. Importantly, the measurements of adipic acid, a non-specific metabolite of DEHA, proved to be important in model calibration.

**Discussion:** The very prominent trends in the urinary excretion of the metabolites, 5cx-MEPA and 5OH-MEHA allowed the important absorption mechanisms of DEHA to be modelled. The model should be useful for the study of exposure to DEHA of the general human population.

## Introduction

Di (2-ethylhexyl) adipate (DEHA; synonyms: bis(2-ethylhexyl) adipate and dioctyladipate (DOA); CAS registry no. 103-23-1; EC no. 203-090-1) is an alternative to the ortho-phthalate plasticizer di (2-ethylhexyl) phthalate (DEHP), which is subject to bans and use restrictions in many countries due to its reproductive toxicity and endocrine disrupting effects (Nehring et al., 2020). DEHA is considered a safe alternative to DEHP. Female rat reproductive system toxicity was observed only at high doses (Dalgaard et al., 2003; Miyata et al., 2006; Wato et al., 2009) and spermatogenesis in male mice was reported to be affected after single intraperitoneal doses of DEHA (Singh et al., 1975). Further, the anti-androgenic effects (Dalgaard et al., 2003; Miyata et al., 2006) and testicular toxicity in rats attributed to DEHP were not observed with DEHA (Kang et al., 2006; Nabae et al., 2006). A tolerable daily intake (TDI) of 0.3 mg/kg bw/day has been set by the European Union Scientific Committee on Food (SCF) (European Commission 2000) and DEHA is listed in Annex I (FCM 207) of Commission Regulation EU No 10/2011 with a specific migration limit (SML) of 18 mg/kg food.

DEHA is used in many different industrial and commercial applications such as flooring and wall coverings, paints and lacquers, polyvinyl chloride (PVC) toys and medical devices as well as in food contact materials in some parts of the world (Silva et al., 2013; Nehring et al., 2020). The migration of DEHA from PVC film into food (Goulas et al., 2008; Fasano et al., 2012) is considered to be a major source of human exposure among the general population (Loftus et al., 1994). In addition, DEHA is present in the aquatic environment (Horn et al., 2004; Barnabé et al., 2008) and in indoor air and dust (Fromme et al., 2016; Subedi et al., 2017; Christia et al., 2019; Giovanoulis et al., 2019) therefore exposure of the general population to DEHA is highly likely.

Human biological monitoring (HBM) is the repeated controlled measurement of a chemical, its metabolites, or biochemical markers in accessible media such as urine, blood and saliva, exhaled air and hair (Manno et al., 2010). As a method of exposure assessment HBM is considered superior to personal air or dermal deposition measurements. This is because more accurate estimates of body burden can be made, since HBM measurements are a composite measure of multiple routes of exposure (Cocker and Jones, 2017). Differences in individual behaviour such as, personal hygiene and work rate, in addition to inter-individual differences in physiology and metabolism can be captured in HBM measurements (Cocker and Jones 2017). Uncertainty in external exposure assessment due to inter- and intra-individual variability can also be reduced by using HBM if the measured biomarker, either parent chemical or metabolite(s), is proportionately related to the ultimate toxic entity (Boogaard et al., 2011). The ability to estimate organ and tissue dose or ‘tissue dosimetry’ from body burdens calculated using HBM should further improve the correlation of exposure to health effects.

Tissue dosimetry can be estimated with the application of physiologically based biokinetic (PBK) modelling. PBK modelling is a powerful means of simulating the factors that determine tissue dose within any biological organism and consequently, it is correlation with health effects (Andersen 1995; Clewell and Andersen 1996; Andersen 2003; Barton et al., 2007; Loizou and Hogg 2011). The value of PBK models is that they are tools for integrating *in vitro*, *in silico* and *in vivo* mechanistic parameters, to simulate the biokinetics of a given chemical and correlate with toxicological information. PBK models encode an explicit mathematical description of important anatomical, physiological, and biochemical determinants of chemical uptake, absorption, distribution, and elimination (ADME). When used in risk assessment, these models can provide a basis for extrapolating between species, doses, and exposure routes or for justifying non-default values for uncertainty factors. Characterization of uncertainty and variability is increasingly recognized as important for risk assessment (Barton et al., 2007). Thus, PBK modelling is increasingly being used in chemical risk assessment (RA) (Chiu et al., 2007; Loizou et al., 2008; WHO 2010).

In this study we apply a PBK model for DEHA based on the model structure for di-(2-propylheptyl) phthalate (DPHP) described previously to interpret the urinary excretion of DEHA metabolites (McNally et al., 2021; McNally and Loizou 2023). The model was parameterized using *in vitro* and *in silico* methods such as, measured intrinsic hepatic clearance scaled from *in vitro* to *in vivo* and predicted octanol–water PC (Log P_ow_) values which, in turn, were used to predict parameters such as plasma unbound fraction and tissue:blood partition coefficients (PCs). Also, global sensitivity analysis (GSA) was used to test the sufficiency and relevance of PBK model structure and the sensitivity of model output to *in vitro* and *in silico* derived model parameters. The latter is part of the ongoing development of a good modelling practice and regulatory acceptance of PBK in chemical safety assessment (Barton et al., 2007; Loizou et al., 2008; Barton et al., 2009; WHO 2010; Paini et al., 2017; Ellison 2018; Fabian et al., 2019; OECD 2021).

## Materials and methods

### Experimental

#### Chemicals

Pooled human microsomes were purchased from Tebu-bio^1^ (Peterborough, UK). The microsomes were prepared from a pool of 50 liver samples, mixed gender (20 mg protein ml^⁻1^). DEHA and the simple monoester mono-2-ethylhexyl adipate (MEHA) (purity >97.4%) were provided by BASF SE. All chemicals used were of analytical grade or higher; B-nicotinamide adenine dinucleotide phosphate (NADP), purity 97%, Glucose-6-phosphate, 98%-100%, Magnesium chloride, ACS reagent >99%, and Glucose-6-phosphate dehydrogenase (type V from baker’s yeast) were obtained from Sigma Aldrich. Potassium dihydrogen phosphate, analytical grade, and Di-potassium hydrogen phosphate, analytical grade, were obtained from Fisher Scientific.

#### Analysis

Samples were analysed by liquid chromatography (Shimadzu Prominence) with tandem mass spectrometry detection (AB Sciex API 3200) using electrospray ionisation. Ion optics, temperatures and gas flows were optimised on our individual system. All analyses used a Synergi Hydro-RP column (150 × 2mm; 4µ; Phenomenex) in conjunction with a methanol:20 mM ammonium acetate (0.1% acetic acid) gradient. Sample injection volume was 2 µl.

#### Determination of *in vitro* and *in vivo* intrinsic clearance

**FIGURE 1:**
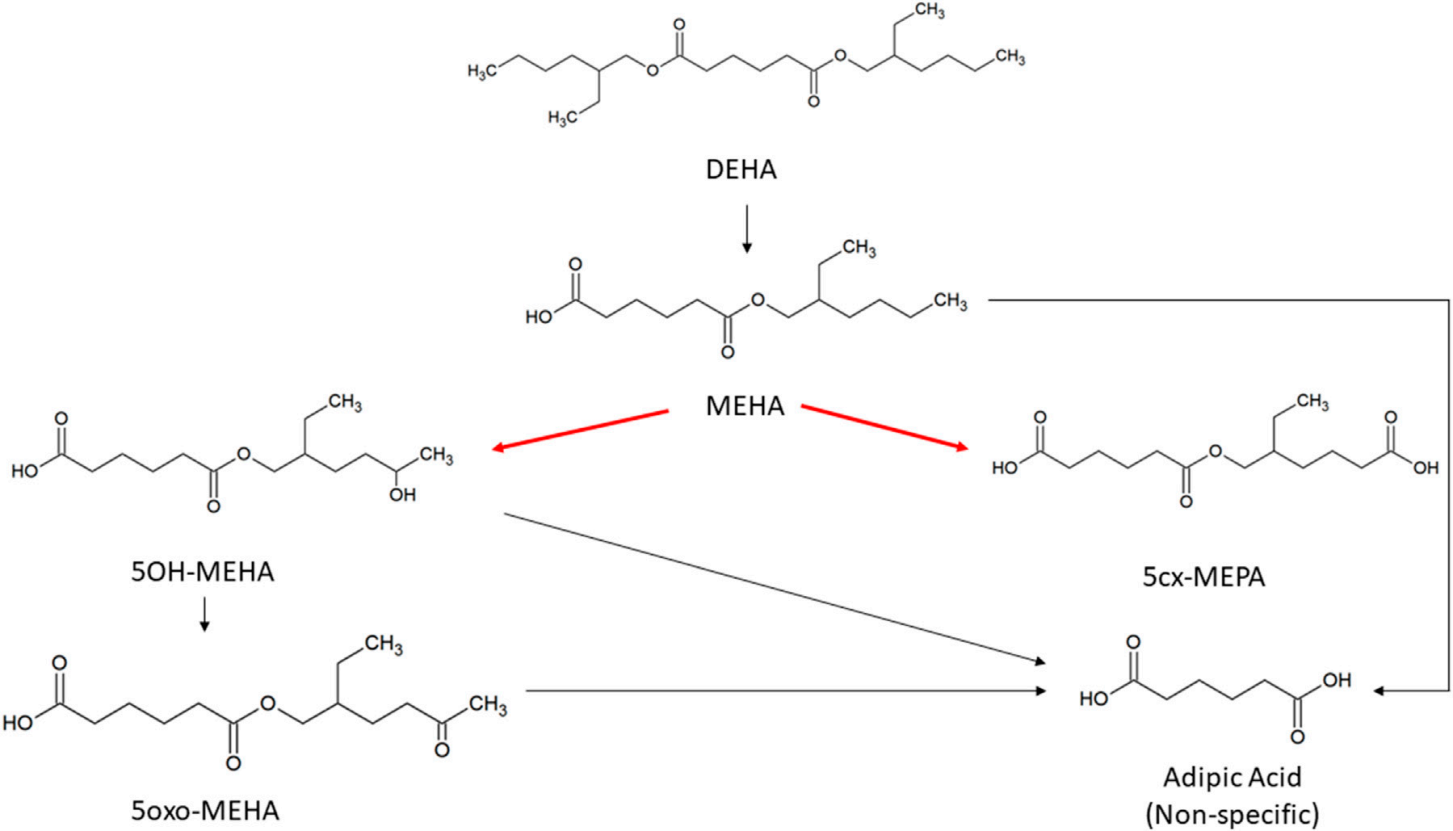
Metabolic pathway of DEHA to the specific, side-chain-oxidized monoesters measured in the controlled human exposure study of (Nehring et al., 2020) and simulated using the PBPK model. The intrinsic clearance, Cl_int_ for the biotransformation of MEHA to the two urinary metabolites is shown by the red arrow. Cleavage to the non-specific metabolite adipic acid (AA), and phase II metabolism (conjugation with, e.g., glucuronic acid) not shown for simplification.

Consistent with previous studies with diisononyl-cyclohexane-1, 2-dicarboxylate (Hexamoll^®^ DINCH) (McNally et al., 2019) and di-(2-propylheptyl) phthalate (DPHP) (McNally et al., 2021; McNally and Loizou 2023) the very high lipophilicity of DEHA resulted in the formation of an insoluble film on the surface of the reaction medium which precluded the measurement of *in vitro* clearance. Therefore, only the measurement of *in vitro* clearance of MEHA was possible (Figure 1). *In vitro* incubations, the determination of *in vitro* half-life, *in vitro* intrinsic clearance and the calculation of *in vivo* clearance were identical to previous studies and are described therein (Pacifici et al., 1988; Soars et al., 2002; Howgate et al., 2006; Barter et al., 2007; McNally et al., 2019; McNally et al., 2021; McNally and Loizou 2023).

#### Prediction of Log P_ow_ and tissue:blood partition coefficients (PCs) and plasma fraction unbound

Tissue:blood PCs and unbound fractions in plasma were calculated from the log of the octanol to water partition coefficient (Log P_ow_) as described in McNally et al. (2019) and McNally et al. (2021). Briefly, the Log P_ow_ for DEHA and MEHA were calculated using the ACDLogP algorithm (Mannhold et al., 2009) implemented in the ACD/ChemSketch 2019.1.0^2^ software (Table 1). The Log P_ow_s were input into two tissue-composition-based algorithms for the calculation of tissue:blood PCs. The method of Poulin and Haddad (2012), which was developed for the prediction of the tissue distribution of highly lipophilic compounds, defined as chemicals with a Log P_ow_ > 5.8, was used for DEHA (Table 1). The method of Schmitt (2008), which was developed to predict the tissue distribution of chemicals with Log P_ow_ < 5.2, was used to predict the PCs of the monoester, MEHA (Table 1). The algorithm of Poulin and Haddad (2012) was implemented as a Microsoft^®^ Excel Add-in whereas a modified version of the algorithm of Schmitt (2008) was available within the httk: R Package for High-Throughput Toxicokinetics (Pearce et al., 2017). Where the tissue-composition-based algorithms did not provide a tissue:blood PC for a particular compartment, the value from a surrogate organ or tissue with similar blood perfusion rate (i.e., could be lumped within the rapidly or slowly perfused compartments) was assumed. These are presented in italicised text with the surrogate organ or tissue in brackets Table 1.

**TABLE 1:** Tissue:blood partition coefficients and plasma fraction unbound predicted using Log P_ow_.

	DEHA	MEHA
Log Po:w	9.54	5.84
Tissue:blood partition coefficient		
Adipose	47.2	2.00
Liver	5.9	10.7
*Muscle*	*3.3*	*1.83*
Blood cells	3.0	1.23
Gut	7.4	3.08
Spleen	3.7	2.56
Stomach^a^ (gut)	7.4	3.08
Rapidly Perfused (spleen)	3.7	2.26
*Slowly Perfused (muscle)*	*3.3*	*1.83*
		
Plasma Fraction Unbound	0.000158	0.007175

^a^ Compartments in italics have surrogate values from another organ compartment. The corresponding surrogate organ compartment is in parentheses.

The fraction unbound (*fu*) was calculated from *log ((1-fu)/fu)* with the following equation:

$$fu=\frac{1}{10^{x}+1}$$ (1)

Where, x=0.4485logP−0.4782

When *x* is the equation for the prediction of *fu* for a chemical with a predominantly uncharged state at pH 7.4 (Lobell and Sivarajah 2003) (Table 1).

#### Biological monitoring data

The biological monitoring (BM) data from the human volunteer study of Nehring et al. (2020) were simulated in this investigation. Briefly, four healthy volunteers (2 females, 2 males; aged between 24 and 34 years; bodyweight between 59 and 91 kg), each received a single oral dose of approximately 10 mg of DEHA, dissolved in 1 ml ethanol and diluted with water, administered in a chocolate coated waffle cup. The resulting individual doses of DEHA ranged from 107 to 164 μg/kg bw. (Table 2). The volunteers (A-D) did not have any known occupational exposure to DEHA. The volunteers donated 22, 26, 21, and 20 individual urine samples over a 48-h period with total urine volumes of 2,800, 5,544, 6,576 and 3,606 ml, respectively.

**TABLE 2:** Volunteer specific parameters.

	A	B	C	D
Body weight (kg)	81	59	91	72
Dose (mg kg^−1^)	0.123	0.169	0.109	0.14
Fraction Metabolised from MEHA				
to 5OH-MEHA	0.0008, 0.00107	0.00045, 0.0163	0.00073, 0.0012	0.00022, 0.00049
to 5cx-MEPA	0.00228, 0.00305	0.0016, 0.00576	0.00205, 0.0033	0.0019, 0.0042
to 5oxo-MEHA	0.0005, 0.00067	0.00048, 0.00173	0.00048, 0.00077	0.00012, 0.00026
To AA	0.742, 0.9952	0.275, 0.991	0.617, 0.995	0.451, 0.995

The concentrations of mono-2-ethyl-5-hydroxyhexyl adipate (5OH-MEHA), mono-2-ethyl-5-oxohexyl adipate (5oxo-MEHA), mono-5-carboxy-2-ethylpentyl adipate (5cx-MEPA), and the non-specific hydrolysis product adipic acid (AA) were extracted from the dataset of (Nehring et al., 2020). Concentrations of AA were only available for the first 24 h of study data. The rates of deposition of these metabolites into the urine (mg/h) were calculated based on the concentrations (mg/l), the volume of the urine void (l) and the time between successive voiding events. This rate represents an average rate of deposition since the previous urination event and renders the trends in urine data more clearly compared to concentrations expressed in (mg/l) or concentrations expressed relative to creatinine (Nehring et al., 2020). The derived rate was associated with the mid-point between the two voiding events.

#### Calculation of fractions metabolised

Whilst it is possible to directly estimate the fractions of *ingested* DEHA eliminated as specific metabolites (5OH-MEHA, 5oxo-MEHA and 5cx-MEPA) from the study of Nehring et al. (2020), for parameterising the PBK model described below it was necessary to 1) estimate the fraction of ingested DEHA that was absorbed and 2) estimate the fractions of *absorbed* DEHA ultimately eliminated as 5OH-MEHA, 5oxo-MEHA and 5cx-MEPA—these fractions are greater than the fractions of *ingested* DEHA eliminated in urine as 5OH-MEHA, 5oxo-MEHA and 5cx-MEPA so long as there is incomplete absorption of DEHA.

The fraction of *ingested* DEHA eliminated as 5OH-MEHA, 5oxo-MEHA and 5cx-MEPA were calculated using Equation 2, where M denotes the molar mass of DEHA and the respective metabolites, D the administered dose (mg) of DEHA and $m_{i}$ denotes the mass of metabolite (mg) deposited in the urine void at time point i . A similar calculation was made for AA but with a correction for background sources. The rate of deposition of AA (mg/h) from background sources was estimated from the final few measurements in the first 24 h of the study (noting that AA was only measured in urine voids within this period). The time points, where the deposition rate of 5cx-MEPA into urine was less than 1% of its maximum value, were used in this calculation, with between 1 and 3 measurement time points used for the four volunteers. The average deposition rate of AA (mg/h) over the selected time points was calculated. A total background deposition of AA (mg) over the 24-h period of the measurements was subsequently calculated.

$$F_{UE}=\frac{1}{D}\sum m_{i}\left(\frac{M\;\left(DEHA\right)}{M\;\left(Metabolite\right)}\right)$$ (2)

A lower bound for the fraction of DEHA absorbed by each volunteer was estimated as the sum of $F_{UE}$ for 5OH-MEHA, 5oxo-MEHA, 5cx-MEPA and AA. The fractions of *absorbed* DEHA eliminated as 5OH-MEHA, 5oxo-MEHA, 5cx-MEPA and AA were calculated as the respective $F_{UE}$ divided by the total. These values are provided in Table 2.

A previous human volunteer study described in Loftus et al. (1993), wherein volunteers were administered deuterium labelled DEHA, estimated that approximately 12% of DEHA was eliminated as non-specific metabolites, principally 2-ethylhexanoic acid (EHA), that were not measured in Nehring et al. (2020). Takahashi et al. (1981) reported that a large fraction of DEHA administered to rats was ultimately exhaled as carbon dioxide. Both Takahashi et al. (1991) and Loftus et al. (1993) reported little faecal excretion. Based on these two studies complete absorption of DEHA was assumed for an upper bound case. Lower bounds on the fractions of absorbed DEHA eliminated as 5OH-MEHA, 5oxo-MEHA, 5cx-MEPA and AA were taken as the respective $F_{UE}$ calculated through (2). These values are also provided in Table 2.

#### The PBK model

An existing human PBK model for DPHP (McNally et al., 2021) was adapted and simplified for studying the absorption, distribution, metabolism, and elimination of DEHA following single oral doses. The DEHA model contained two important simplifications compared to that of DPHP - a single rather than a two-phased gut compartment, and the removal of the coding of enterohepatic recirculation - therefore testing of the PBK model for DEHA, sufficient to verify the coding of the model and its ability to capture the trends seen in the BM data of (Nehring et al., 2020) has been undertaken.

**FIGURE 2:**
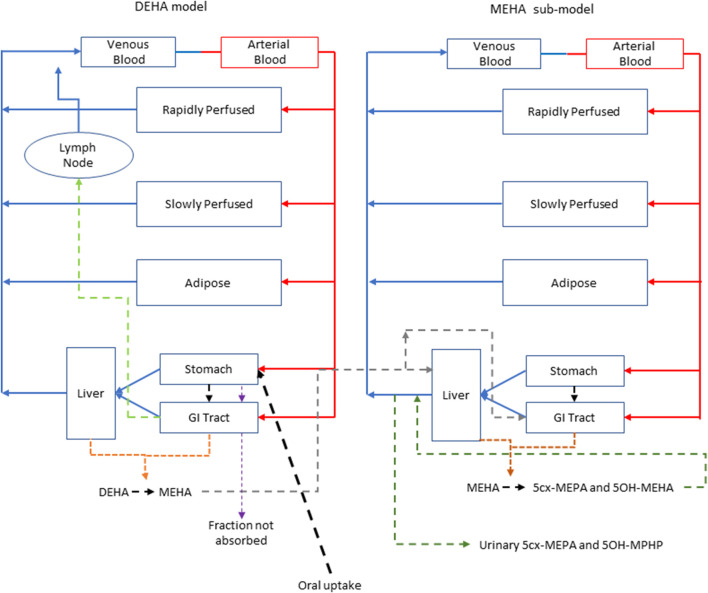
A schema of the model for DEHTP and sub-model for MEHTP. The main model contained a lymphatic compartment (- - - -) which received a portion of oral dose from the stomach and GI tract. Urinary excretion of metabolites was described with a first-order elimination rate constant ascribed to the sub-model.

Briefly, the model for DEHA described two distinct uptake processes and allowed for a fraction to pass directly through the gut and be ultimately eliminated in faeces (Figure 2). The first uptake process was into blood. The model included a description of absorption of DEHA from the stomach and gastro-intestinal (GI) tract. The second important uptake mechanism of DEHA was into the lymphatic system. Uptake of DEHA *via* the lacteals in the intestine and entering venous blood after bypassing the liver was coded - a delay function (Lymphlag) was coded to account for the relatively slow transportation of DEHA through the lymphatic system. Inclusion of a lymph compartment was based on the assumption that DEHA, like DEHP, binds like lipid to lipoproteins (Griffiths et al., 1988) which are formed in enterocytes and transported in the lymph to enter the venous blood *via* the thoracic duct (Kessler et al., 2012). The fractions of dose entering venous blood, the lymphatic system and passing straight through the gut summed to unity.

The model for DEHA had stomach and gut compartments draining into the liver and systemically circulated to adipose, blood (plasma and red blood cell) and slowly and rapidly perfused compartments. Protein binding was described in arterial blood, with only the unbound fraction of DEHA available for distribution to organs and tissues and metabolism. Metabolism of DEHA to MEHA was ascribed to the liver and the gut.

A sub-model was coded to describe the kinetics of MEHA. As described above, metabolism of DEHA to MEHA was coded in the gut and the liver, therefore models for DEHA and MEHA were connected at these nodes in the model. Metabolism of MEHA was coded in the liver alone. The MEHA sub-model had a stomach (Loizou and Spendiff 2004) and intestine draining into the liver and systemically circulated to adipose, blood (plasma and red blood cell) and slowly and rapidly perfused compartments. As with the DEHA model, binding was described in arterial blood.

To make use of biological monitoring data from the human volunteer study of Nehring et al. (2020) it was necessary to describe further metabolism of MEHA in the PBK model. As indicated in earlier discussion, the metabolic pathways of DEHA are non-trivial and downstream metabolites of DEHA may be ultimately eliminated in both urine and exhaled breath. A simplified representation of metabolism of MEHA was assessed as being suitable for the aims of the study with the focus on two immediate metabolites of MEHA, 5OH-MEHA and 5cx-MEPA, eliminated in urine. Amounts of 5OH-MEHA and 5cx-MEPA produced were expressed as fractions of metabolised MEHA, and these were eliminated from blood into urine at a rate proportional to the amount in blood. First order elimination constants described the removal of these respective fractions from blood and into urine. The kinetics of these second order metabolites were thus described using four parameters in all; the model did not describe the distribution of these metabolites to organs and tissues. Whilst neither data on 5oxo-MEHA nor background corrected AA were ultimately used (Table 2), these data were indirectly utilised in forming appropriate ranges for fractions of MEHA metabolised to and eliminated from blood as 5OH-MEHA and 5cx-MEPA.

The model code is available in Supplementary Material.

### Statistical analysis

#### Parameter distributions

Probability distributions for uncertainty and sensitivity analysis of the final PBK model are listed in Table 3. Anatomical and physiological parameter distributions were obtained from the freely available web-based application PopGen (McNally et al., 2014). A population of 10,000 individuals comprising 50% Caucasian males and 50% Caucasian females was generated. The range of ages, heights and body weights supplied as input to PopGen were chosen to encompass the characteristics of the volunteers who participated in the human volunteer study (Nehring et al., 2020). Parameter ranges for organ masses and blood flows were modelled by normal or log-normal distributions as appropriate with parameters estimated from the sample and truncated at the 5th and 95th percentiles.

**TABLE 3:** Physiological and kinetic default values used in PBPK model and probability distributions applied for uncertainty and sensitivity analyses. Physiological and kinetic constants used in PBPK model.

Physiological parameters	Abbreviation	Default value	Distribution
Body weight (kg)	BW	89	N^1^ (49, 130)
% BW			
Total vascularised tissues	VT	0.95	-
Liver	VLiC	3.09	N (3.09, 0.8)
Fat	VFaC	19.5	LN (3.42, 0.43)
Gut	VGuC	1.50	U (1.19, 1.84)
Stomach	VStC	0.22	N (0.22, 0.07)
Slowly perfused tissue	VSpdC	60.7	N (60.7, 9.4)
Rapidly perfused tissue	VRpdC	3.71	N (3.7, 0.26)
Blood	VBldC	5.0	N (5, 1)
Cardiac output (L h^−1^ kg^−1^ BW)	QCC	14	N (13.8, 2.5)
% Cardiac output			
Liver	QHepartC	6.0	N (6.89, 0.52)
Fat	QFaC	5.0	N (5.3, 0.3)
Gut	QGuC	14.9	U (13.2,16.6)
Stomach	QStC	1.1	N (1.1, 0.08)
Slowly perfused tissue	QSpdC	27.0	N (28.7, 1.91)
Rapidly perfused tissue	QRpdC	42.0	N (43.1, 2.78)
Metabolic Clearance (minutes)			
*In vivo* half-life DEHA	T_½DEHA_	3^2^	U (15, 60)
*In vitro* half-life MEHA	T_½MEHA_	22.8	N (22.8, 3)
*In vivo* DEHA gut half-life	T_½DEHA_gut_	60^3^	U (15, 60)
Microsomal protein yield (mg g^−1^)			
Hepatic	MPY	34^4^	See Table 4
Gut	MPY_gut_	3.9^5^	U (1.95, 7.8)
Blood:tissue partition coefficients			
*DEHA*			
Plasma	Pbab	3.0	U (1,50)
Adipose	Pfab	47.2	U (1,50)
Liver	Plib	5.9	U (1,50)
Gut	Pgub	7.4	U (1,50)
Stomach	Pstb	7.4	U (1,50)
Rapidly Perfused	Prpdb	7.4	U (1,50)
Slowly Perfused	Pspdb	3.3	U (1,50)
MEHA			U (1,50)
Plasma	PbaM	1.23	U (1,50)
Adipose	PfaM	2.00	U (1,50)
Liver	PliM	10.7	U (1,50)
Gut	PguM	3.08	U (1,50)
Stomach	PstM	3.08	U (1,50)
Rapidly Perfused	PrpdM	2.26	U (1,50)
Slowly Perfused	PspdM	1.83	U (1,50)
Blood:tissue partition coefficients			
*DEHTP*			
Plasma	Pbab	15.5	U (1,30)
Adipose	Pfab	47.2	U (32, 125)
Liver	Plib	5.89	U (1,50)
Kidney	Pkib	3.7	U (3, 12)
Red blood cells	Prbcb	3.0	U (1, 10)
Gut	Pgub	7.4	U (1,50)
Stomach	Pstb	3.7	U (2, 8)
Rapidly Perfused	Prpdb	3.7	U (2, 8)
Slowly Perfused	Pspdb	3.3	U (2,8)
*MEHTP*			
Plasma	PbaM	25.23	U (1, 50)
Adipose	PfaM	20.3	U (15, 60)
Liver	PliM	5.9	U (1, 30)
Kidney	PkiM	12.2	U (1, 30)
Red blood cells	PrbcM	6.67	U (3, 12)
Gut	PguM	7.4	U (1, 30)
Stomach	PstM	7.4	U (12, 50)
Rapidly Perfused	PrpdM	3.7	U (6, 24)
Slowly Perfused	PspdM	3.3	U (4, 15)

Fraction bound in plasma (proportion)	Abbreviation	Default value	Distribution
DEHA	FBDEHA		U (0.8, 1)
MEHA	FBMEHA		U (0.8, 1)
Gastric emptying (h^−1^)^6^			
Maximum	k_(max)_	10.2	U (5.1, 20.4)
Minimum	k_(min)_	0.005	U (0.0025, 0.01)
Absorption (h^−1^)			
Gut	k_Ga_	25.1	U (12.55, 50.2)
Time taken to consume dose (h)	DRINKTIME	0.25	U (0.125, 0.5)
Absorption in Stomach	BELLYPERM	0.685	U (0.05, 7.5)
Absorption in GI Tract	GIPERM	5.1	U (0.05, 30)
Absorption in Lymph *via* stomach	BELLYPERMLymph	0.685	U (0.34, 0.99)
Absorption in Lymph *via* GI Tract	GIPERMLymph	5.1	U (2.6, 7.6)
Absorption into blood from lymph	K1Lymph	0.2	U (0.05, 5)
Delay associated with lymphatic uptake	LymphLag	1	(0.05, 5)
Absorbed fraction of DEHA	FracAbsorbed	0.5	U (0.25, 1)
Fraction of dose taken up into liver	FRACDOSEHep	0.5	U (0, 1)
Fraction of MEHA to 5OH-MEHA	FracMetabOH		U (0.0004, 0.002)
Fraction of MEHA to 5cx-MEPA	FracMetabcx		U (0.0015, 0.006)
Urinary elimination rate (h^−1^)			
5OH-MEHA	K1_MOH	1	U (0.5, 5)
5cx-MEPA	K1_5cx	1	U (0.5, 5)

Uniform distributions were ascribed to the various delay terms and uptake and elimination rates. The upper and lower bounds in Table 3 were refined during the model development process. The tabulated values are therefore based upon expert judgement and represent conservative yet credible bounding estimates.

#### Uncertainty and sensitivity analysis

McNally et al. (2021) describe an interactive approach for development and testing of the human PBK model for DPHP using techniques for uncertainty and sensitivity analysis to study the behaviour of the model and the key parameters that drove variability in the model outputs. The principal techniques used for model evaluation were Latin Hypercube Sampling (LHS), to evaluate the qualitative behaviour of the model, and a two-phased sensitivity analysis consisting of elementary effects screening and a variance-based sensitivity analysis to identify the important uncertain parameters in the model to be refined in calibration.

As described previously the DEHA model contained two important simplifications compared to that of DPHP - a single rather than a two-phased gut compartment, and the removal of the coding of enterohepatic recirculation - therefore testing of the PBK model for DEHA, sufficient to verify the coding of the model and its ability to capture the trends seen in the BM data of (Nehring et al., 2020) has been undertaken.

In the first phase of analysis a 200-point Latin Hypercube Design (LHD) was used to draw a sample of parameter sets that efficiently explored the parameter space defined by the parameter distributions given in Table 3. For each of these *design points* the PBK model was run and data from four outputs—concentrations of DEHA and MEHA (mg/l) in blood, and rates of deposition of 5OH-MEHA and 5cx-MEPA into urine (mg/h) were extracted. The concentration-time profiles over the design points were used to visually assess the bounding behaviour of the model (model form coupled with parameter distributions) and assess whether the model was broadly consistent with trends in the volunteer BM data.

Sensitivity analysis using elementary effects screening was subsequently applied to determine the subset of sensitive parameters to take forward to calibration. A total of 52 parameters were varied with seven elementary effects per model parameter computed, leading to a design of 371 runs of the PBK model. The ranges for each parameter in elementary effects screening were derived from the 2.5th and 97.5^th^ percentiles for the respective probability distributions (Table 3). The Morris test was applied to the model outputs of: DEHA and MEHA concentrations in venous blood (mg/l) at 0.5- and 5-h following ingestion of DEHA; and for rates of deposition of 5OH-MEHA and 5cx-MEPA into the bladder (mg/h) at 1-, 3-, 5- and 10-h following ingestion of DEHA. Euclidean distance from the origin was computed from the Morris Test output for each parameter, with parameter rankings at each time point based upon this measure. The results were normalised at each time point such that a value of unity corresponded to the most important parameter at a given time point.

The initial filter for further consideration of a parameter to be taken forward into calibration was a normalised Euclidean distance in excess of 0.1 for at least one of the twelve measures. A final subset of sensitive parameters was obtained following a further phase of review.

#### Calibration

Calibration is the process of tuning a subset of model parameters such that the discrepancy between model predictions and comparable measurement data is minimised. This is achieved through the specification of an error model that links predictions to measurements. A Bayesian approach to calibration was followed (McNally et al., 2012) as this allows the uncertainty in the concentration-response predicted by the PBK model, which is a function of a subset of sensitive model parameters, to be explicitly quantified.

A Bayesian approach requires the specification of a joint prior distribution for the parameters under study. It is necessary to distinguish between two classes of parameters: global parameters which are common to all individuals, which are appropriate for various constants and physicochemical properties such as partition coefficients etc; local parameters, which vary between individuals are suitable for accounting for variability in the physiology and modelling the participant specific uptake of DEHA etc. These two classes of model parameters are denoted by the vectors θ and $\omega_{j}$ respectively, where the subscript *j = 1 … 4,* denotes the participant. A prior distribution for each global parameter was specified through the distributions provided in Table 3. A prior distribution for each individual, four copies in all, was specified for each of the local parameters. These distributions are also provided in Table 3. A median and 95% credible interval for global and local parameters is provided in Tables 4 (global) and Table 5 (locals) respectively.

**TABLE 4:** Global prior and posterior distributions.

Parameter	Median (95% interval)	
	Prior	Posterior
FB_DEHA	0.900 (0.805, 0.995)	0.848 (0.802, 0.935)
FB_MEHA	0.900 (0.805, 0.995)	0.871 (0.803, 0.973)
DEHA_GUT_half_life	30.02 (10.82, 49.39)	26.94 (4.60, 48.20)
DEHA_half_life	20.30 (0.933, 67.19)	3.19 (0.10, 17.84)
MEHA_half_life	22.8 (16.99, 28.622)	20.65 (15.76, 26.60)
Pbab	15.49 (1.70, 29.24)	23.31 (9.69, 29.77)
Pgub	25.47 (2.13, 48.73)	13.39 (3.89, 27.79)
Plib	25.47 (2.13, 48.73)	23.42 (2.09, 48.34)
PbaM	25.47 (2.13, 48.73)	34.64 (9.25, 49.23)
PliM	15.49 (1.70, 29.24)	16.37 (2.29, 29.18)
PguM	15.49 (1.70, 29.24)	8.24 (1.45, 23.84)
K1_cx	2.72 (0.622, 4.90)	1.54 (1.14, 2.11)
K1_OH	2.72 (0.622, 4.90)	4.31 (2.97.4.96)
FracMetab_cx	0.00375 (0.0016, 0.0059)	0.00039 (0.00030, 0.00051)
FracMetab_OH	0.00089 (0.0002, 0.0016)	0.00018 (0.00014, 0.00023)
$\sigma_{cx\_U}$	0.67 (0.032, 2.20)	0.0004 (0.00, 0.00052)
$\sigma_{OH\_U}$	0.67 (0.032, 2.20)	0.00018 (0.00013, 0.00023)

**TABLE 5:** Local posterior distributions.

Parameter	Prior	Ind A	Ind B	Ind C	Ind D
FracAbsorbed	0.627 (0.269, 0.983)	0.857 (0.647, 0.991)	0.534 (0.390, 0.752)	0.776 (0.584, 0.971)	0.396 (0.266, 0.597)
FracDOSEHep	0.503 (0.023, 0.973)	0.381 (0.318, 0.478)	0.624 (0.511, 0.753)	0.411 (0.328, 0.515)	0.323 (0.221, 0.457)
BELLYPERM	3.72 (0.24, 7.30)	1.52 (0.12, 6.16)	4.53 (0.45, 7.36)	3.95 (0.253, 7.32)	3.49 (0.25, 7.26)
GIPERM	14.79 (0.77, 29.26)	18.25 (6.65, 29.37)	4.68 (1.91, 25.57)	0.806 (0.481, 1.45)	16.31 (4.54, 29.21)
Lymphlag	2.99 (0.15, 5.87)	1.87 (1.66, 2.09)	4.09 (3.57, 4.64)	3.84 (3.63, 3.99)	4.10 (3.72, 4.68)
K1_Lymph	2.54 (0.177, 4.87)	1.33 (0.91, 2.63)	2.93 (1.14, 4.88)	3.40 (1.69, 4.89)	3.13 (0.97, 4.92)
MPY	34.0 (14.54, 53.77)	40.25 (24.30, 57.75)	32.09 (13.16, 52.05)	39.56 (21.77, 57.77)	35.05 (18.00, 53.69)
VBldC	0.05 (0.031, 0.070)	0.05 (0.032, 0.070)	0.05 (0.031, 0.069)	0.049 (0.03, 0.069)	0.049 (0.031, 0.069)
VliC	0.03 (0.011, 0.05)	0.034 (0.019, 0.048)	0.034 (0.016, 0.048)	0.033 (0.017, 0.048)	0.031 (0.015, 0.047)
VguC	0.015 (0.010, 0.020)	0.014 (0.010, 0.019)	0.016 (0.011, 0.019)	0.015 (0.011, 0.019)	0.015 (0.011, 0.019)
QguC	0.150 (0.089, 0.21)	0.178 (0.127, 0.230)	0.149 (0.094, 0.207)	0.182 (0.132, 0.234)	0.156 (0.103, 0.212)

The second facet of model specification is the statistical error model. The final calibration model utilised HBM data from the four volunteers with two specific outputs formally compared within the error model. The rates of deposition of 5OH-MEHA and 5cx-MEPA (mg/h) into the urine (*RUrine OH* and *RUrine cx*) were computed from the raw data of Nehring et al. (2020) as described earlier, and compared with equivalent predictions extracted from the PBK model through Equations 3, 4.

The terms ${RUrineOH}_{ij}$ and ${RUrinecx}_{ij}$ denote measurement i (at time $t_{i}$ ) for individual j (for j *in 1:4)* for the two respective model outputs, whereas $\mu_{OH\_U}{\left(\theta ,\omega_{j}\right)}_{ij}$ and $\mu_{cx\_U}{\left(\theta ,\omega_{j}\right)}_{ij}$ , denote the predictions from the PBK model corresponding to parameters ( $\theta ,\omega_{j}$ ). Normal distributions, truncated at zero were assumed for both these relationships, where $\sigma_{OH\_U}$ and $\sigma_{cx\_U}$ denote the respective error standard deviations.

$${RUrineOH}_{ij}∼\;N\left(\mu_{OH\_U}{\left(\theta ,\omega_{j}\right)}_{ij},\sigma_{OH\_U}\right)\left[0,\infty \right]$$ (3)

$${RUrinecx}_{ij}∼\;N\left(\mu_{cx\_U}{\left(\theta ,\omega_{j}\right)}_{ij},\sigma_{cx\_U}\right)\left[0,\infty \right]$$ (4)

Weakly informative, half-normal prior distributions with standard deviations of 1 were assumed for the standard deviation parameters in Equations 2, 3.

Inference for the model parameters was made using Markov chain Monte Carlo (MCMC) implemented in MCSim (see Software). Inference for model parameters in the final calibration model was made using thermodynamic integration (TI) as described in Bois et al. (2020). A single chain of 150,000 iterations was run with every 10th retained.

#### Software

The PBK model was written in the GNU MCSim^3^ language and run using the RStudio Version 1.3.1093^4^. The DiceDesign package of R^5^. was used for generating Latin Hypercube designs. GSA of model outputs through elementary effects screening was conducted using the Sensitivity package of R. The reshape2 package of R was used for reshaping of data for plotting and other processing of results. MCMC was undertaken using the thermodynamic integration (TI) option within GNU MCSim. All plots were created using R and the ggplot2^6^ package.

## Results

### Uncertainty and sensitivity analyses

**FIGURE 3:**
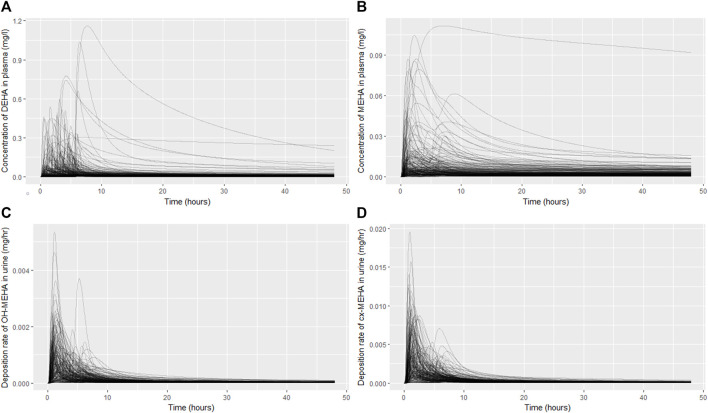
Uncertainty analysis of the concentration response profiles in venous blood (mg/l) for DEHA **(A)** and MEHA **(B)**, and the urinary excretion (mg/h) of 5OH-MEHA **(C)** and 5cx-MEPA **(D)**.

The results from uncertainty analysis are shown in Figure 3. Panel 4A shows the concentration response profiles for DEHA in venous blood (mg/l), Panel 4B shows the concentration response profiles for MEHA in venous blood (mg/l), Panel 4C shows the concentration response profiles for urinary excretion of 5OH-MEHA (mg/h) and Panel 4D shows the concentration response profiles for urinary excretion of 5cx-MEPA (mg/h). The profiles in the figure indicate a wide range of behaviour for the four outputs under study and was consistent with the model form and probability distributions ascribed to the model parameters, particularly so for the simulations of DEHA and MEHA concentrations in blood, where peak concentrations and the rate of removal varied substantially over the design points. There was no data available for direct comparison against these two measures, however it is useful to study concentrations since refinement based upon expert knowledge with reference to similar chemicals may assist in refining parameter ranges. The simulations of the urinary excretion of 5OH-MEHA and 5cx-MEPA (mg/h) showed lower variability compared to panels A) and B), with all curves showing the appearance of a very rapid peak and a decay in deposition rate toward zero over the period of the simulation. A subset of curves showed a bimodal profile within varying time periods between peaks. This initial cursory comparison of the range of model behaviour relative to the distinct trends seen for the four volunteers suggested that the model was sufficiently flexible to simulate the available biological monitoring data.

Results from sensitivity analysis for the 12 measures under study are given in Supplementary Table S1 of Supplementary Material. Parameters with a normalised Euclidean distance of greater than 0.10 for any of the measures are highlighted in bold.

Following a review of the results from elementary effects screening twenty-six parameters (15 global and 11 local) (Tables 4, 5) were taken forward into calibration.

### Calibration

**FIGURE 4:**
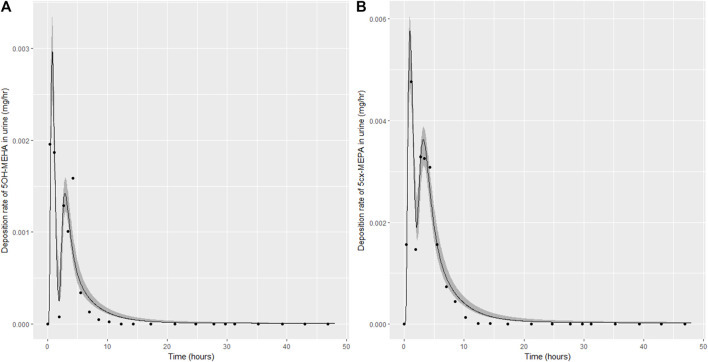
Simulation of the urinary excretion (mg/h) of 5OH-MEHA **(A)** and 5cx-MEPA **(B)** for volunteer A.

**FIGURE 5:**
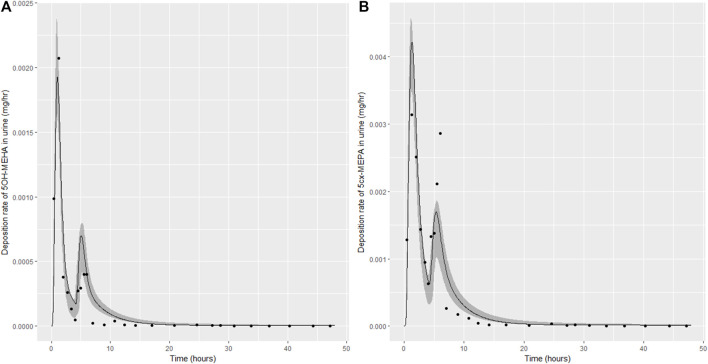
Simulation of the urinary excretion (mg/h) of 5OH-MEHA **(A)** and 5cx-MEPA **(B)** for volunteer B.

**FIGURE 6:**
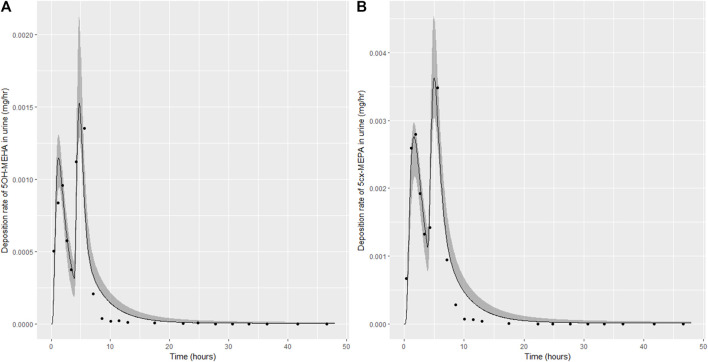
Simulation of the urinary excretion (mg/h) of 5OH-MEHA **(A)** and 5cx-MEPA **(B)** for volunteer C.

**FIGURE 7:**
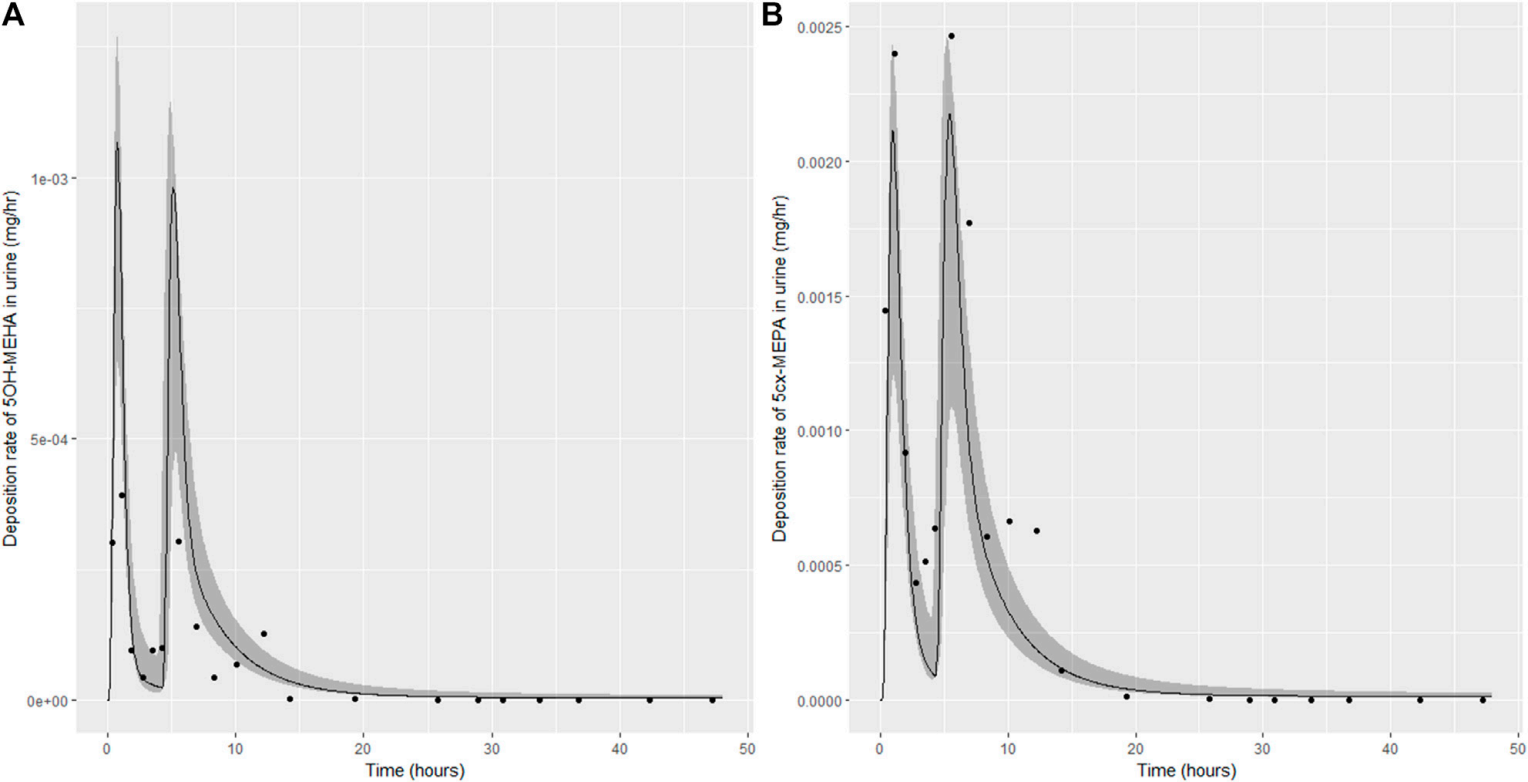
Simulation of the urinary excretion (mg/h) of 5OH-MEHA **(A)** and 5cx-MEPA **(B)** for volunteer D.

Summary statistics based upon the retained sample (posterior median and a 95% credible interval) for the 15 global and 11 local (volunteer specific) parameters are provided in Tables 4, 5 respectively. The fit of the calibrated model is shown in Figures 4–7 for individuals A, B, C and D, respectively. A comparison of the calculated (based upon the posterior mode) and measured 48-h excretions of 5cx-MEPA and 5OH-MEHA in urine is provided in Table 6.

**TABLE 6:** Comparisons of predictions and measured 48-h eliminations of 5OH-MEHA and 5cx-MEPA for the four volunteers under the posterior mode parameter set.

	5OH-MEHA		5cx-MEPA	
	Measured	Predicted	Measured	Predicted
V1	0.006972	0.00763	0.01808	0.02207
V2	0.003933	0.00510	0.01272	0.01477
V3	0.006347	0.00658	0.01629	0.01906
V4	0.001937	0.00354	0.01493	0.01026

The two panels in each figure correspond to A) deposition of 5OH-MEHA in urine (mg/h); B) deposition of 5cx-MEPA in urine (mg/h). The central estimates (solid line) indicated in plots correspond to the posterior mode parameter set, the single best fitting parameter set over the 8 measures (2 outputs for each of 4 individuals) used for calibration. The shaded regions represent pointwise 95% credible intervals for the respective curves. This interval was derived by running each retained sample drawn from the posterior through the PBK model and storing the output from each model output from 0 to 48 h in 0.05- hour increments. Output at each time point was retained and ordered with the 2.5th and 97.5th percentiles saved; the plotted 2.5% and 97.5% bounds are a smooth interpolation of these series of pointwise values.

The HBM data from the four volunteers, expressed as rates of deposition into urine (mg/h), each showed very strong bimodal profiles (Figures 4–7), although with variations between the volunteers in the relative magnitudes of the peaks and in the time duration between the two modes. The fits demonstrate that the PBK model generally provided a good fit to the measurements, although the model did not capture the second peak of 5cx-MEPA for individual B (Figure 5) and the very rapid declines in deposition rates of 5OH-MEHA and 5cx-MEPA following the second peak (in the period more than 5 h following ingestion) could not be simulated for some volunteers—as a consequence there was a tendency to over-predict the 48-h excretions of 5cx-MEPA and 5OH-MEHA in urine although these were generally within 20% of the measured values. Based on the overall quality of fit, despite simplifications, the PBK adequately describes the key mechanisms to describe the appearance of 5cx-MEPA and 5OH-MEHA in urine.

In the model coding, a fraction absorbed was coded with the limits of the prior distribution based upon the fractions of ingested DEHA excreted in urine as four downstream metabolites of DEHA in urine (a uniform prior distribution U (0.25, 1) was specified). There was a substantial narrowing of this relatively wide range for each volunteer with estimates of fractions absorbed of 0.827 (0.647, 0.991), 0.534 (0.390, 0.752), 0.776 (0.584, 0.971) and 0.396 (0.266, 0.597) for volunteers A to D respectively. There was also a very considerable narrowing for the parameter FracDOSEHep (the fraction of absorbed DEHA entering *via* the hepatic route with the complementary fraction entering *via* the lymphatic system) for each volunteer relative to the U (0, 1) prior. The hepatic fraction was estimated as 0.381 (0.318, 0.478), 0.642 (0.511, 0.753), 0.411 (0.328, 0.515) and 0.323 (0.221, 0.457) for individuals A to D respectively. Whilst this varied substantially over the volunteers, results suggest both routes are important, with approx. 1/3 and 2/3 of absorbed DEHA entering *via* hepatic and lymphatic routes respectively.

## Discussion

In this work we have presented the first available PBK model for DEHA. The structure of the model was based on the previously published PBK model for DPHP (McNally et al., 2021; McNally and Loizou, 2022 in press) and initial model parameterisation based upon *in silico* and *in-vitro* experimental data. Several simplifications were subsequently made to the model form - principally the removal of enterohepatic recirculation and reversion to a single-phase intestine. Global sensitivity analysis is recognised as an important tool in model development and testing (McNally et al., 2011; Loizou et al., 2015; Lumen et al., 2015) and this was conducted using LHS to efficiently assess the overall behaviour of the model, and with elementary effects screening to flag important sensitive parameters to be taken forward into calibration. Whilst variance-based methods are generally accepted as providing the ‘gold standard’ for global sensitivity analysis, the results from this class of methods are influenced by both the sensitivity of model output to changes in parameters *and* the probability distributions ascribed to those uncertain parameters. Given the significant uncertainty associated with many parameters, represented in this work with uniform distributions with wide ranges, the choice of probability distributions ascribed to model parameters could have had an undue influence of the results of the GSA in a variance-based analysis. The more computationally efficient elementary effects screening (Morris Test) provides lower precision measures of sensitivity; however, it requires only ranges rather than probability distributions to be specified; it was judged as being a more appropriate methodology for the analysis of this model.

Nehring et al. (2020) identified specific metabolites of DEHA that could be used to infer population exposures to DEHA based upon concentrations in spot urine voids (under certain assumptions). The total fractions of ingested DEHA eliminated as these specific metabolites could be estimated from the study data and are sufficient for interpreting data from human biomonitoring. However, these specific metabolites only account for ∼0.5% of the ingested chemical. The measurements of AA in the HBM study (Nehring et al., 2020) proved to be important in calibrating the PBK model by allowing us to specify prior distributions that better constrained the absorbed fraction. Previous work has suggested that DEHA has complex metabolic pathways with a further five non-specific metabolites, accounting for a mean of 12% of administered DEHA (Loftus et al., 1993). Whilst these metabolites were not specifically accounted for in the model, some account was taken of them, albeit weakly through the lower bounds on the prior distributions of FracMetab_cx and FracMetab_OH and the upper bound on FracAbsorbed. The very prominent trends of 5cx-MEPA and 5OH-MEHA observed in the urine of the study participants has allowed the important absorption mechanisms of DEHA to be modelled and following calibration we may estimate reasonable probability bounds on the appearance of DEHA and MEHA in blood and organs and tissues. However, the data from the BM study of Nehring et al. (2020) are only weakly informative on the suite of metabolic products of DEHA.

In previous human (Loftus et al., 1993) and animal studies (Takahashi et al., 1981) with DEHA, the authors noted there was little faecal excretion of DEHA. Under an assumption of near complete absorption of DEHA, the large differences between volunteers from the HBM study of Nehring et al. (2020), the largest being between volunteers A and D, would have to arise due to excretion mechanisms that are not accounted for within the model. The ultimate elimination of absorbed DEHA through respiratory CO_2_ has been suggested by Loftus et al. (1993) and Takahashi et al. (1981). In the rat study of Takahashi et al. (1981) the ^14^C-radioactivity in exhaled breath was used to estimate excretion fractions of ∼40% and 60% as exhaled CO_2_ in two rats. Whilst this magnitude of between-participant variability appears to be consistent with the human volunteer study of Nehring et al. (2020) some caution is required in assuming complete absorption; studies with other plasticizers such as Hexamoll^®^ DINCH (Koch et al., 2013) and DPHP (Klein et al., 2018) have demonstrated that the ‘ingestion vehicle’ for the parent chemical can influence absorption. In principle, the elimination of DEHA in exhaled breath could be included in an extended PBK model, however more HBM studies would be required to describe the exhaled chemical and the full metabolic pathway. Whilst of some scientific interest, the costs of additional research in this area would likely only be justified by specific safety concerns.

## Data Availability

The original contributions presented in the study are included in the article/Supplementary Material, further inquiries can be directed to the corresponding author.

## Footnotes

1
https://www.tebu-bio.com/ (14/02/2023).

2
https://www.acdlabs.com/.

3
https://www.gnu.org/software/mcsim/ (as on 05/10/2022).

4
https://www.rstudio.com/products/rstudio/download/ (as on 05/10/2022).

5
https://www.r-project.org/ (as on 05/10/2022).
