# Supplementary material for: Development, testing, parameterisation, and calibration of a human PBK model for the plasticiser, di (2-ethylhexyl) adipate (DEHA) using in silico, in vitro and human biomonitoring data
Source: Front Pharmacol. 2023 Mar 23;14:1165770. doi: 10.3389/fphar.2023.1165770 (PMC10076754; doi:10.3389/fphar.2023.1165770)
Supplement: Supplementary file 1 [file Table7.DOCX]

Supplementary material to *Development,* testing, parameterisatio*n and calibration of a human PBPK model for the plasticiser, Di (2-ethylhexyl) adipate (DEHA) using in silico, in vitro and human bio-monitoring data*

Kevin McNally^1^, Craig Sams^1^, and George Loizou^1^

Author’s affiliation

^1^Health and Safety Executive, Harpur Hill, Buxton UK

Table S1: Normalised Euclidean distances for model parameters, calculated from elementary effects screening output, computed for each of the 12 metrics under study. All parameters with a normalised Euclidean distance in excess of 0.1 for at least one measure are highlighted in bold

| parameters | DEHA_0.5 | DEHA_5.0 | MEHA_0.5 | MEHA_5.0 | OH_1.0 | OH_3.0 | OH_5.0 | OH_10.0 | cx_1.0 | cx_3.0 | cx_5.0 | cx_10.0 |
| --- | --- | --- | --- | --- | --- | --- | --- | --- | --- | --- | --- | --- |
| BW | 0 | 0 | 0 | 0 | 0 | 0 | 0 | 0 | 0 | 0 | 0 | 0 |
| **VliC** | 0.038 | 0.007 | 0.09 | 0.02 | 0.078 | 0.12 | 0.038 | 0.147 | 0.069 | 0.095 | 0.04 | 0.198 |
| **VfaC** | 0.061 | 0.057 | 0.077 | 0.006 | 0.042 | 0.126 | 0.032 | 0.115 | 0.041 | 0.146 | 0.059 | 0.081 |
| **VguC** | 0.073 | 0.006 | 0.347 | 0.009 | 0.2 | 0.225 | 0.068 | 0.082 | 0.198 | 0.242 | 0.083 | 0.088 |
| VstC | 0.014 | 0.005 | 0.036 | 0.006 | 0.046 | 0.092 | 0.025 | 0.102 | 0.036 | 0.028 | 0.025 | 0.039 |
| **VspdC** | 0.052 | 0.166 | 0.163 | 0.356 | 0.02 | 0.133 | 0.021 | 0.047 | 0.021 | 0.103 | 0.021 | 0.055 |
| VrpdC | 0.005 | 0.006 | 0.008 | 0.02 | 0.001 | 0.03 | 0.006 | 0.015 | 0.002 | 0.046 | 0.006 | 0.025 |
| **VBldC** | 0.026 | 0.348 | 0.065 | 0.1 | 0.002 | 0.019 | 0.007 | 0.016 | 0.003 | 0.042 | 0.008 | 0.017 |
| QCC | 0.021 | 0.014 | 0.056 | 0.019 | 0.043 | 0.061 | 0.016 | 0.053 | 0.044 | 0.026 | 0.017 | 0.048 |
| **QhepartC** | 0.005 | 0.004 | 0.1 | 0.105 | 0.012 | 0.032 | 0.005 | 0.024 | 0.014 | 0.034 | 0.005 | 0.026 |
| QfaC | 0.006 | 0.005 | 0.014 | 0.009 | 0.003 | 0.017 | 0.008 | 0.041 | 0.006 | 0.015 | 0.01 | 0.044 |
| **QguC** | 0.058 | 0.046 | 0.131 | 0.108 | 0.125 | 0.376 | 0.04 | 0.049 | 0.131 | 0.289 | 0.035 | 0.06 |
| QstC | 0.024 | 0.005 | 0.034 | 0.018 | 0.067 | 0.031 | 0.028 | 0.05 | 0.054 | 0.022 | 0.027 | 0.047 |
| **QspdC** | 0.017 | 0.007 | 0.051 | 0.01 | 0.01 | 0.027 | 0.029 | 0.108 | 0.005 | 0.048 | 0.032 | 0.141 |
| **QrpdC** | 0.028 | 0.023 | 0.039 | 0.043 | 0.008 | 0.139 | 0.012 | 0.101 | 0.035 | 0.071 | 0.023 | 0.132 |
| **DEHA_GUT_half_life** | 0.023 | 0.078 | 0.126 | 0.012 | 0.293 | 0.123 | 0.026 | 0.054 | 0.016 | 0.109 | 0.033 | 0.142 |
| **DEHA_half_life** | 0.041 | 0.049 | 0.148 | 0.073 | 0.31 | 0.109 | 0.035 | 0.041 | 0.221 | 0.09 | 0.029 | 0.076 |
| **MEHA_half_life** | 0 | 0 | 0.189 | 0.444 | 0.227 | 0.292 | 0.014 | 0.05 | 0.273 | 0.211 | 0.028 | 0.099 |
| **MPY** | 0.051 | 0.044 | 0.173 | 0.074 | 0.673 | 0.153 | 0.086 | 0.16 | 0.356 | 0.146 | 0.06 | 0.293 |
| MPYgu | 0.006 | 0.007 | 0.029 | 0.005 | 0.034 | 0.044 | 0.012 | 0.033 | 0.074 | 0.039 | 0.011 | 0.042 |
| **FB_DEHA** | 1 | 1 | 0.124 | 0.379 | 0.022 | 0.952 | 1 | 0.688 | 0.021 | 1 | 1 | 0.662 |
| **FB_MEHA** | 0 | 0 | 1 | 1 | 0.036 | 0.088 | 0.064 | 0.286 | 0.048 | 0.087 | 0.067 | 0.319 |
| **KEMAX** | 0.171 | 0.04 | 0.125 | 0.006 | 0.022 | 0.034 | 0.007 | 0.033 | 0.027 | 0.022 | 0.006 | 0.032 |
| KEMIN | 0.001 | 0 | 0 | 0 | 0.001 | 0.002 | 0 | 0.002 | 0.001 | 0.001 | 0 | 0.002 |
| KA_MEHA | 0 | 0 | 0 | 0 | 0 | 0 | 0 | 0 | 0 | 0 | 0 | 0 |
| DRINKTIME | 0.07 | 0.003 | 0.094 | 0.002 | 0.123 | 0.049 | 0.008 | 0.007 | 0.081 | 0.044 | 0.006 | 0.007 |
| **BELLYPERM** | 0.016 | 0.017 | 0.146 | 0.045 | 0.083 | 0.209 | 0.065 | 0.064 | 0.079 | 0.143 | 0.056 | 0.062 |
| **GIPERM1** | 0.841 | 0.348 | 0.732 | 0.01 | 0.117 | 0.295 | 0.042 | 0.107 | 0.135 | 0.198 | 0.031 | 0.108 |
| BELLYPERMlymph | 0 | 0 | 0 | 0 | 0 | 0 | 0 | 0 | 0 | 0 | 0 | 0 |
| GIPERMlymph | 0.002 | 0 | 0 | 0 | 0.001 | 0.002 | 0.001 | 0 | 0.002 | 0.001 | 0.002 | 0.001 |

Table S1 continued: Normalised Euclidean distances for model parameters, calculated from elementary effects screening output, computed for each of the 12 metrics under study. All parameters with a normalised Euclidean distance in excess of 0.1 for at least one measure are highlighted in bold

| parameters | DEHA_0.5 | DEHA_5.0 | MEHA_0.5 | MEHA_5.0 | OH_1.0 | OH_3.0 | OH_5.0 | OH_10.0 | cx_1.0 | cx_3.0 | cx_5.0 | cx_10.0 |
| --- | --- | --- | --- | --- | --- | --- | --- | --- | --- | --- | --- | --- |
| **K1Lymph** | 0 | 0.206 | 0 | 0.002 | 0 | 0.239 | 0.048 | 0.103 | 0 | 0.204 | 0.089 | 0.164 |
| **Lymphlag** | 0.36 | 0.215 | 0.001 | 0.018 | 0.002 | 0.556 | 0.25 | 0.637 | 0.005 | 0.576 | 0.231 | 0.637 |
| **FracAbsorbed** | 0.156 | 0.188 | 0.655 | 0.255 | 0.826 | 0.757 | 0.185 | 0.437 | 0.803 | 0.486 | 0.158 | 1 |
| **FracDOSEHep** | 0.364 | 0.908 | 0.383 | 0.831 | 1 | 0.945 | 0.225 | 0.499 | 1 | 0.829 | 0.262 | 0.749 |
| **FracMetabMOH** | 0 | 0 | 0 | 0 | 0.714 | 1 | 0.881 | 1 | 0 | 0 | 0 | 0 |
| **FracMetabcx** | 0 | 0 | 0 | 0 | 0 | 0 | 0 | 0 | 0.782 | 0.748 | 0.744 | 0.848 |
| **K1_MOH** | 0 | 0 | 0 | 0 | 0.195 | 0.243 | 0.072 | 0.072 | 0 | 0 | 0 | 0 |
| **K1_cx** | 0 | 0 | 0 | 0 | 0 | 0 | 0 | 0 | 0.22 | 0.249 | 0.077 | 0.276 |
| Pfab | 0 | 0 | 0 | 0 | 0 | 0.008 | 0.008 | 0.044 | 0 | 0.003 | 0.006 | 0.045 |
| **Plib** | 0.08 | 0.005 | 0.134 | 0.024 | 0.036 | 0.084 | 0.018 | 0.06 | 0.045 | 0.046 | 0.021 | 0.069 |
| **Pgub** | 0.012 | 0.001 | 0.35 | 0.024 | 0.134 | 0.173 | 0.035 | 0.029 | 0.209 | 0.061 | 0.045 | 0.028 |
| Pstb | 0.035 | 0.003 | 0.033 | 0.003 | 0.031 | 0.038 | 0.015 | 0.028 | 0.033 | 0.049 | 0.014 | 0.024 |
| Prpdb | 0.002 | 0.003 | 0 | 0.002 | 0.001 | 0.297 | 0.061 | 0.128 | 0.002 | 0.185 | 0.045 | 0.073 |
| **Pspdb** | 0 | 0.013 | 0 | 0.014 | 0.001 | 0.025 | 0.07 | 0.476 | 0.001 | 0.039 | 0.132 | 0.413 |
| **Pbab** | 0.009 | 0.065 | 0.052 | 0.04 | 0.098 | 0.039 | 0.021 | 0.33 | 0.105 | 0.047 | 0.026 | 0.377 |
| PfaM | 0 | 0 | 0.001 | 0.002 | 0 | 0.008 | 0.008 | 0.065 | 0 | 0.006 | 0.009 | 0.062 |
| **PliM** | 0 | 0 | 0.612 | 0.014 | 0.096 | 0.132 | 0.034 | 0.095 | 0.226 | 0.167 | 0.043 | 0.105 |
| **PguM** | 0 | 0 | 0.025 | 0.006 | 0.098 | 0.164 | 0.08 | 0.123 | 0.085 | 0.054 | 0.072 | 0.117 |
| PstM | 0 | 0 | 0.001 | 0 | 0.001 | 0.002 | 0.001 | 0.004 | 0.001 | 0.002 | 0.001 | 0.005 |
| PrpdM | 0 | 0 | 0.07 | 0.004 | 0.014 | 0.004 | 0.005 | 0.014 | 0.016 | 0.009 | 0.009 | 0.023 |
| PspdM | 0 | 0 | 0.004 | 0.015 | 0.001 | 0.015 | 0.012 | 0.092 | 0.003 | 0.077 | 0.028 | 0.128 |
| PbaM | 0 | 0 | 0.02 | 0.073 | 0.029 | 0.075 | 0.006 | 0.015 | 0.041 | 0.116 | 0.017 | 0.034 |

PBPK model code for the DEHA .model file

# MCSim 6.1.0 model of Di(2-ethylhexyl) adipate

# Compiled on 06/10/2022.

States =

{

Afa,

Agu,

AMgu,

Ast,

Arpd,

Aspd,

Ali,

AMli,

ABile,

Alymph,

ARBC_DEHA,

Aplasm_DEHA,

ABellyH,

AGiTractH,

ABowel,

ABellylymph,

AGiTractlymph,

AfaM,

AstM,

AguM,

AMliM,

AliM,

ABileM,

ABowelM,

AspdM,

ArpdM,

Aplasm_MEHA,

ARBC_MEHA,

AMMEHAB_MOH,

AMMEHAB_cx,

AMMEHAU_MOH,

AMMEHAU_cx,

VBladder,

Lymphswitch,

DOSESTEP

};

Outputs =

{

Cli,

Cfa,

Cgu,

Cst,

Clymph,

Cspd,

Crpd,

mass,

Uptake,

reloral,

CVfa,

CVgu,

CVst,

CVspd,

CVli,

CVrpd,

CV,

CVnmol,

CA_DEHA,

CAT_DEHA,

CARBC_DEHA,

CV_total_nmol,

CVM,

CliM,

CVliM,

CguM,

CVguM,

CstM,

CVstM,

CfaM,

CVfaM,

CspdM,

CVspdM,

CrpdM,

CVrpdM,

CA_MEHA,

CRBC_MEHA,

Curine_MOH,

Curine_cx,

ODOSEliver,

ODOSElymph,

ODOSEbowel,

ClintDEHA,

ClintDEHAgu,

ClintMEHA,

Ali_lag,

AliM_lag,

Blood_DEHA,

Blood_MEHA,

Urine_cx,

Urine_OH

};

Inputs =

{

events_Lymphswitch,

events_DOSESTEP,

events_VBladder,

events_AMMEHAU_MOH,

events_AMMEHAU_cx

};

# Parameters

BW = 89; # body mass (kg)

MWDEHA = 370.574; # DEHA molecular mass (g/mol)

MWMEHA = 258.35; # MEHA molecular mass (g/mol)

MWMEHAOH = 322.39; # OH-MEHA molecular mass (g/mol)

MWMEHAcx = 294.34; # cx-MEHA molecular mass (g/mol)

CAE = 0.75; # cardiac allometric exponent

QCC = 11.22; # cardiac allometric constant (L/h/kg^CAE)

VT = 0.95; # proportion of vascularised tissue

VfaC = 0.195; # fractional volume

VguC = 0.067; # fractional volume

VstC = 0.0158; # fractional volume

VspdC = 0.4714; # fractional volume poorly perfused

VrpdC = 0.033; # fractional volume richly perfused

VliC = 0.0203; # fractional volume

VlymphC = 0.0036; # lymph system fractional volume

VBldC = 0.05; # blood fractional volume

QhepartC = 0.06; # hepatic artery fractional blood flow

QguC = 0.17; # fractional blood flow

QstC = 0.01; # fractional blood flow

QspdC = 0.27; # overall fractional blood flow to slowly perfused tissue

QrpdC = 0.42; # overall fractional blood flow to rapidly perfused tissue

QfaC = 0.05; # fractional blood flow

FracAbsorbed = 0.5; # Fraction of dose taken into lymph

FracDOSEHep = 0.5; # Fraction of dose taken into hepatic

FracMetabMOH = 0.01; # Fraction of CYP-mediated metabolism MEHA -> MEHA Arch Tox 87)

FracMetabcx = 0.01; # Fraction of CYP-mediated metabolism MEHA -> MEHA

FB_DEHA = 0.9975; # Fraction of DEHA bound to plasma proteins

FB_MEHA = 0.9854; # Fraction of MEHA bound to plasma proteins

PORALDOSE = 0.7; # oral dose [mg/kg]

DRINKTIME = 0.05; # Drink time [h]

BELLYPERM = 0.685; # [/h]

GIPERM1 = 25.1; # [/h]

BELLYPERMlymph = 0.685; # [/h]

GIPERMlymph = 5.1; # [/h]

KEMAX = 10.2; # [Maximum emptying rate /h]

KEMIN = 0.005; # [Minimum emptying rate /h]

KA_MEHA = 0.3; # 1st-order oral uptake rate of MEHA (1/hr)

Lymphswitch = 1;

MPY = 34; # microsomal protein yield [mg microsomal protein/g liver]

MPYgu = 3.9; # microsomal protein yield [mg microsomal protein/g gut]

Incub_vol = 1; # Volume of incubation (ml)

Microsome_prot = 0.5; # microsomal protein amount (mg)

DEHA_half_life = 3; # DEHA -> MEHA half-life (minutes)

DEHA_GUT_half_life = 60; # DEHA -> MEHA GUT half-life (minutes)

MEHA_half_life = 20; # MEHA -> OH-MEHA and cx-MEHA half-life (minutes)

RUrine = 0.1; # Rate of Urine Production [l/h]

Creat = 1.217; # Urinary creatinine concentration [g/L] or 0.01192 [mol/L]

K1_MOH = 1; # First-order elimination rate from blood [/h]

K1_cx = 1; # First-order elimination rate from blood [/h]

K1_DEHA_LIVER = 1; # First-order elimination rate of MEHA from liver into bile [/h]

K1_MEHA_LIVER = 1; # First-order elimination rate of MEHA from liver into bile [/h]

K1Lymph = 0.2; # First-order elimination rate from Lyph into blood [/h]

Lymphlag = 3.01; # Lag between uptake into Lymph and emptying into blood [h]

Pbab = 3.01; # DEHA blood:air partition coefficient

Pfab = 63.38; # DEHA tissue:blood partition coefficient

Pgub = 7.4; # DEHA tissue:blood partition coefficient

Pstb = 7.4; # DEHA tissue:blood partition coefficient

Prpdb = 3.7; # DEHA tissue:blood partition coefficient

Pspdb = 3.29; # DEHA tissue:blood partition coefficient

Plib = 5.89; # DEHA tissue:blood partition coefficient

PbaM = 6.67; # MEHA Red blood cells:plasma partition coefficient

PspdM = 7.51; # MEHA Slowly perfused tissue:air partition coefficient

PliM = 54.8; # MEHA tissue:air partition coefficient

PrpdM = 12.20; # MEHA Richly tissue:air partition coefficient

PfaM = 29.10; # MEHA Fat tissue:air partition coefficient

PstM = 25.2; # MEHA Stomach tissue:air partition coefficient

PguM = 25.2; # MEHA GI Tract tissue:blood partition coefficient

Vfa = 0;

Vgu = 0;

Vst = 0;

Vspd = 0;

Vrpd = 0;

Vli = 0;

Vlymph = 0;

Qfa = 0;

Qgu = 0;

Qst = 0;

Qrpd = 0;

Qspd = 0;

Qli = 0;

QCMC = 0;

ODOSEliver = 0;

ODOSElymph = 0;

ODOSEbowel = 0;

Uptake = 0;

Vplas = 0;

VRB = 0;

Qhepart = 0;

CA_DEHA = 0;

CVnmol = 0;

CVM = 0;

escapeFrac = 0;

# SD terms for MCMC

Sigma1 = 0.1;

Sigma2 = 0.1;

Initialize

{

BWc = pow(BW, CAE); # cardiac scaling output factor (kg)

VplasC = 0.55 * VBldC; # plasma fractional volume

HEME = 1 - (VplasC / VBldC); # Volume of Haeme

VRBC = HEME * VBldC; # Volume of red blood cells

## Gelman reparameterisations

Qcci = QrpdC + QspdC + QhepartC + QfaC + QstC + QguC;

Qrpdci = QrpdC / Qcci;

Qspdci = QspdC / Qcci;

Qhepartci = QhepartC / Qcci;

Qfaci = QfaC / Qcci;

Qstci = QstC / Qcci;

Qguci = QguC / Qcci;

Vti =

(1 - VT) + VrpdC + VspdC + VliC + VfaC + VstC + VguC + VplasC + VRBC + VlymphC;

Vguci = VguC / Vti;

Vstci = VstC / Vti;

Vfaci = VfaC / Vti;

Vlici = VliC / Vti;

Vspdci = VspdC / Vti;

Vrpdci = VrpdC / Vti;

Vbldci = VBldC / Vti;

Vplasci = VplasC / Vti;

VRBCci = VRBC / Vti;

Vlymphci = VlymphC / Vti;

# Volumes scaled to actual volumes

Vfa = Vfaci * BW; # scaled fractional volume

Vgu = Vguci * BW; # scaled fractional volume

Vst = Vstci * BW; # scaled fractional volume

Vspd = Vspdci * BW; # scaled fractional volume

Vli = Vlici * BW; # scaled fractional volume

Vrpd = Vrpdci * BW; # scaled fractional volume

Vlymph = Vlymphci * BW; # scaled fractional volume

VRB = VRBCci * BW; # scaled red blood cell fractional volume

#Vplasci = 0.55 * VBldC; # plasma fractional volume

Vplas = Vplasci * BW; # plasma fractional volume

# Calculate actual blood flows from total flow and percent flows

QC = QCC * BWc; # cardiac output (L/h)

Qfa = Qfaci * QC; # scaled fractional blood flow

Qgu = Qguci * QC; # scaled fractional blood flow

Qst = Qstci * QC; # scaled fractional blood flow

Qrpd = Qrpdci * QC; # scaled fractional blood flow

Qspd = Qspdci * QC; # scaled fractional blood flow

Qhepart = Qhepartci * QC; # scaled hepatic artery fractional blood flow

Qli = Qhepart + Qst + Qgu; # scaled fractional blood flow

QCMC = Qhepart + Qgu + Qst + Qfa + Qrpd + Qspd;

} # End of model initialization

Dynamics

{

tau = 4; # the required delay

Ali_lag = CalcDelay(Ali, tau);

AliM_lag = CalcDelay(AliM, tau);

ORALDOSE = PORALDOSE * BW; # scaled oral dose (mg/day)

DOSEFLOW = ORALDOSE / DRINKTIME; # zero order uptake rate constant

ODOSE = DOSEFLOW * DOSESTEP; # amount absorbed (mg)

ODOSEliver = ODOSE * FracAbsorbed * FracDOSEHep;

ODOSElymph = ODOSE * FracAbsorbed * (1 - FracDOSEHep);

ODOSEbowel = ODOSE * (1 - FracAbsorbed);

ClintDEHA = (0.693 / DEHA_half_life) * (Incub_vol / Microsome_prot) * MPY * Vli * 60; # Clearance (L/h whole liver)

ClintDEHAgu = (0.693 / DEHA_GUT_half_life) * (Incub_vol / Microsome_prot) * MPYgu * Vgu * 60; # Clearance (L/h gut)

ClintMEHA = (0.693 / MEHA_half_life) * (Incub_vol / Microsome_prot) * MPY * Vli * 60; # Clearance (L/h whole liver)

# DEHA Concentrations in Compartments

# cellular concentrations (mg/L)

Cfa = Afa / Vfa;

Cgu = Agu / Vgu;

Cst = Ast / Vst;

Cspd = Aspd / Vspd;

Crpd = Arpd / Vrpd;

Cli = Ali / Vli;

Clymph = Alymph / Vlymph;

# venous organ concentration (mg/L)

CVfa = Cfa / Pfab;

CVgu = Cgu / Pgub;

CVst = Cst / Pstb;

CVspd = Cspd / Pspdb;

CVli = Cli / Plib;

CVrpd = Crpd / Prpdb;

GPER = KEMAX / (1 + KEMIN * Cst);

# venous concentration (mg/L)

CV = ((CVfa * Qfa) + (CVrpd * Qrpd) + (CVspd * Qspd) + (CVli * Qli)) / QCMC;

# DEHA Venous concentration (nmoles/L)

CVnmol = (CV / MWDEHA) * 1000000;

# Fraction unbound

Aplasmub_DEHA = Aplasm_DEHA * (1 - FB_DEHA);

mass =

ARBC_DEHA + Aplasm_DEHA + AMli + Ali + ABile + AMgu + ABellyH + AGiTractH + Ast + Agu + ABowel + ABellylymph + AGiTractlymph + Alymph + Afa + Arpd + Aspd; # mass in system (kg)

Uptake = ODOSEliver + ODOSElymph + ODOSEbowel;

# mass balance

reloral = ((t>0) ? mass / (ORALDOSE + 1e-10) : 1);

CA_DEHA = Aplasmub_DEHA /Vplas; # Arterial unbound concentration (nmol/L)

CRBC_DEHA = ARBC_DEHA/VRB; # Concentration in red blood cells (nmol/L)

# MEHA Concentrations in Compartments

# cellular concentrations (mg/L)

CguM = AguM / Vgu;

CstM = AstM / Vst;

CfaM = AfaM / Vfa;

CliM = AliM / Vli;

CspdM = AspdM / Vspd;

CrpdM = ArpdM / Vrpd;

# venous organ concentration (mg/L)

CVguM = CguM / PguM;

CVstM = CstM / PstM;

CVfaM = CfaM / PfaM;

CVliM = CliM / PliM;

CVspdM = CspdM / PspdM;

CVrpdM = CrpdM / PrpdM;

CVM =

((CVliM * Qli) +

(CVfaM * Qfa) +

(CVspdM * Qspd) +

(CVrpdM * Qrpd)) / QCMC;

#unbound model

Aplasmub_MEHA = Aplasm_MEHA * (1 - FB_MEHA);

CA_MEHA = Aplasmub_MEHA / Vplas;

CRBC_MEHA = ARBC_MEHA / VRB;

# DEHA Differential Equations

dt (Lymphswitch) = 0;

dt (DOSESTEP) = 0;

dt (VBladder) = RUrine;

dt (ARBC_DEHA) = (CA_DEHA - CRBC_DEHA / Pbab); # Amount in red blood cells

dt (Aplasm_DEHA) = QCMC * (CV - CA_DEHA) - dt (ARBC_DEHA) + Lymphswitch * Alymph * K1Lymph;

# Amount in plasma

dt (AMli) = ((Qli * ClintDEHA) / (Qli + ClintDEHA / Pbab)) * CVli; # Amount of hepatic metabolism

dt (Ali) = (Qhepart * CA_DEHA) + (Qst * CVst) + (Qgu * CVgu) - (Qli * CVli) - dt (AMli) - (K1_DEHA_LIVER * Ali);

# Amount in liver

dt (ABile) = K1_DEHA_LIVER * (Ali - Ali_lag); # Amount in bile

dt (AMgu) = ((Qgu * ClintDEHAgu) / (Qgu + ClintDEHAgu / Pbab)) * CVgu; # Amount of gut metabolism (

dt (ABellyH) = (ODOSEliver) - (GPER * ABellyH) - (BELLYPERM * ABellyH); # Amount in stomach lumen(mg)

dt (AGiTractH) = (GPER * ABellyH) - (GIPERM1 * AGiTractH); # Amount in GI Tract lumen

dt (Ast) = Qst * (CA_DEHA - CVst) + BELLYPERM * ABellyH; # Amount in stomach compartment

dt (Agu) = Qgu * (CA_DEHA - CVgu) + (GIPERM1 * AGiTractH) - dt (AMgu) + (K1_DEHA_LIVER * Ali_lag);

# Amount in gut compartment

dt (ABowel) = ODOSEbowel ; # Elimination from gut into faeces

dt (ABellylymph) = (ODOSElymph) - (GPER * ABellylymph) - (BELLYPERMlymph * ABellylymph);

# DEHA rate of uptake in lymph compartment

dt (AGiTractlymph) = (GPER * ABellylymph) - (GIPERMlymph * AGiTractlymph);

# DEHA rate of uptake in lymph compartment

dt (Alymph) = (BELLYPERMlymph * ABellylymph) + (GIPERMlymph * AGiTractlymph) -

Lymphswitch * Alymph * K1Lymph; # Amount in lymph

dt (Afa) = Qfa * (CA_DEHA - CVfa); # Amount in fat

dt (Arpd) = Qrpd * (CA_DEHA - CVrpd); #Amount in RPD

dt (Aspd) = Qspd * (CA_DEHA - CVspd); # Amount in SPD

# MEHA Differential Equations

dt (AMliM) = ((Qli * ClintMEHA) / (Qli + ClintMEHA / PbaM)) * CVliM;

dt (AliM) = (Qhepart * CA_MEHA) + (Qst * CVstM) + (Qgu * CVguM) - (Qli * CVliM) + dt (AMli) -

dt (AMliM) - (K1_MEHA_LIVER * AliM); # Amount in liver

dt (ABileM) = K1_MEHA_LIVER * (AliM - AliM_lag); # Amount in bile

dt (AstM) = Qst * (CA_MEHA - CVstM); #Amount in stomach

dt (AguM) = Qgu * (CA_MEHA - CVguM) + ((1 - escapeFrac) * dt (AMgu)) + (K1_MEHA_LIVER * AliM_lag);

#Amount in gut

dt (ABowelM) = 0; Elimination from gut into faeces

dt (AfaM) = Qfa * (CA_MEHA - CVfaM); #Amount in fat

dt (AspdM) = Qspd * (CA_MEHA - CVspdM); #Amount in SPD

dt (ArpdM) = Qrpd * (CA_MEHA - CVrpdM); #Amount in RPD

dt (ARBC_MEHA) = (CA_MEHA - CRBC_MEHA / PbaM); #Amount in red blood cells

dt (Aplasm_MEHA) = QCMC * (CVM - CA_MEHA) - dt (ARBC_MEHA) + (escapeFrac * dt (AMgu));

#Amount in plasma

# MEHA Urinary excretion

dt (AMMEHAB_MOH) =

dt (AMliM) * FracMetabMOH * (MWMEHA / MWDEHA) * (MWMEHAOH / MWMEHA) -

(K1_MOH * AMMEHAB_MOH);

dt (AMMEHAB_cx) =

dt (AMliM) * FracMetabcx * (MWMEHA / MWDEHA) * (MWMEHAcx / MWMEHA) -

(K1_cx * AMMEHAB_cx);

dt (AMMEHAU_MOH) = K1_MOH * AMMEHAB_MOH;

dt (AMMEHAU_cx) = K1_cx * AMMEHAB_cx;

Curine_MOH = K1_MOH * AMMEHAB_MOH;

Curine_cx = K1_cx * AMMEHAB_cx;

}

CalcOutputs

{

CAT_DEHA = Aplasm_DEHA/(MWDEHA*Vplas)*1e6; # Total concentration in plasma (nmol/l)

CARBC_DEHA = ARBC_DEHA / (MWDEHA*VRB)*1e6; # Total concentration in RBC (nmol/l)

CV_total_nmol = (CAT_DEHA + CARBC_DEHA); # Total concentration in blood (nmol/l)

Blood_DEHA = (Aplasm_DEHA + ARBC_DEHA)/(VRB + Vplas); # Total concentration DEHA in blood (mg/l)

Blood_MEHA = (Aplasm_MEHA + ARBC_MEHA)/(VRB + Vplas) * (MWMEHA / MWDEHA); # Total concentration MEHA in blood (mg/l)

Urine_cx = AMMEHAB_cx*K1_cx; #Rate of deposition of cx in urine (ug/h)

Urine_OH = AMMEHAB_MOH*K1_MOH; # #Rate of deposition of OH in urine (ug/h)

}

End.
